# The Principles of Surgery

**Published:** 1845-06

**Authors:** 


### BIBLIOGRAPHICAL NOTICES.

*The Principles of Surgery.* By JOHN MILLER, Professor of Surgery in the University of Edinburgh, &c., &c. Philadelphia: Lee & Blanchard, 1845, p. 524. (From the Publishers.)

This work has been republished in this country without the usual "notes and additions," and heralded simply by a very modest preface, in which the Author states, that it is "intended to exhibit a condensed view of the Principles of the Healing Art," and that the pages "contain the substance of the Author's Lectures on this subject." It may not be known to many of our readers that he is the successor of Sir Charles Bell, and from the examination we have been able to give the work, as well from the reception it has met with, it goes far to show that the station of that celebrated man has not been filled by an unworthy successor. The work is essentially one of *principles*, clear and concise, embracing an excellent view of the science in its present state, but conservative in its character, and does not favor many of the theories and operations which have been more or less in vogue in many countries, for several years past.

The name of the publishing house is a sufficient guarantee for the execution of the work. Instead of giving an analysis of its contents, we present our readers with the following observations on Neuralgia of Joints. p. 305

*Neuralgia of Joints.*—Affections of joints, dependent on inflammatory action and the structural changes thereby induced, are the most frequent in occurrence. We are, however, not without examples of local irritation, in which perverted vascular action is almost wholly in abeyance. The prominent characteristic is pain, unaccompanied by swelling, or other indication of structural change. The affection may be either primary, constituting a disease per se; or it may be secondary, merely a symptom of an earlier and more grave disorder. In the knee, for example, we may have nervous pain, either as a symptom of morbus coxarius, or a truly neuralgic affection of that part, independent of disease elsewhere—although, indeed, the last observation must be made with some reservation, inasmuch as there are found but few cases of neuralgia, in that or any other joint, which are not more or less connected with a perverted state, as to structure, function, or both, in some of the internal organs.

Neuralgic affection of the joints is characterized by a class of symptoms sufficiently distinct; a circumstance of much importance, inasmuch as the appropriate treatment is very different from that which is demanded for structural change. The pain has the ordinary character of the nervous; remittent, intermittent, not slowly and steadily increasing, not constant, not increased by pressure, and not limited to one part, but diffused over the whole of a wide extent of surface. The patient's mind may be diverted from the uneasiness, by conversation, or otherwise engaging the attention; and while the mind is so occupied, the pain is really absent. There is no swelling; at least if there be, it is but trivial in all respects; a mere puffiness, by œdema of the surface; not at all resembling what follows inflammatory action in any part of the textures of the joint. Motion is well borne; and so is manipulation, even rude; the uneasy sensations are not increased by either. The joint itself may be jarred, pressed, jerked with impunity; whereas much complaint may follow a pinching of the super-imposed integument; that texture sometimes seeming to be of greatly increased sensibility. There is no flexion of the joint, as in serious structural change; on the contrary, the limb will most frequently be found extended. The spasms too are wanting, which so frequently attend and invariably aggravate acute vascular disease. The patient is obviously out of health; and labours under irritation, general as well as local; but the system is uninvolved in either inflammatory or hectic fever.

This affection more frequently occurs in females than in males. And usually the symptoms will be found at least connected, if not caused, by disorder of an internal organ; hysteria; dyspepsia; irritation of the bowels, by worms, or by lodgment of other noxious matter. In children, some affections of the joints, apparently neuralgic, would seem to depend on the irritation of dentition.

The *treatment* of neuralgic joints is mainly directed towards the general system; restoring normal functions to the uterus, stomach and intestines, as the circumstances of the case may require. The local applications need be but simple. The serious treatment for structural change would here be not only unnecessary, but certain to prove injurious. The endermoid use of nitrate of silver, so as merely to blacken the surface, is on the whole the preferable application; it not only is really efficient towards the mitigation of the neuralgy, but also, having an imposing character in the eyes of the patient, is useful by satisfying the mental anxiety, which always attends, and sometimes is not the least prominent of the symptoms. Medicated friction or fomentation may also prove of service in a similar manner. But every stimulus; at all powerful, should be either abstained from, or most cautiously used; inasmuch as the morbid condition of the nervous system of the part may here as elswhere, prove but a stepping-stone towards the accession of inflammatory action, entailing serious structural change.

The vital importance of a careful diagnosis need not be insisted on. Lest, on the one hand, we treat with unwarrantable severity a comparatively trifling disorder; and on the other hand, lest we commit the greater error, of supposing a really formidable change of structure in bone, cartilage, or synovial membrane, to be but a nervous affection, and discover not our error until loss of texture and function has become not only great but wholly irremediable.

